# When does ChatGPT help or hinder? A scoping review of influencing factors in college EFL learners' affective and cognitive experiences

**DOI:** 10.3389/fpsyg.2026.1949431

**Published:** 2026-09-29

**Authors:** Ronglin Fu

**Affiliations:** College of Foreign Languages and International Education, Shanghai Jian Qiao University, Shanghai, China

**Keywords:** affective experience, ChatGPT, cognitive experience, English as a foreign language, influencing factors

## Abstract

Existing reviews have established the overall direction of ChatGPT's effects on college English as a Foreign Language (EFL) learners' affective and cognitive experiences but have not systematically addressed under what conditions these effects arise. This scoping review maps the influencing factors reported in 26 empirical studies examining ChatGPT and closely related LLM-based generative AI tools as shaping these experiences. A systematic search of Web of Science, Scopus, and ERIC following PRISMA-ScR guidelines and the (Arksey and O'malley, 2005) framework yielded 829 records, with 26 studies meeting inclusion criteria after deduplication and two-stage screening. Influencing factors were extracted from each study and organized into learner-level, instructional-level, and technology-level categories based on the Cognitive-Affective Theory of Learning with Media. Ten types of influencing factors were identified. Evidence density varied markedly across the three levels, with learner-level factors supported by the largest body of research and technology-level factors by the smallest. Self-efficacy showed consistent mediating pathways across eight studies, while metacognitive awareness produced the most clearly documented conditional differentiation between strategic and surface-level ChatGPT use. Structured instructional integration was the factor with the most consistently positive effects across the three studies that examined it. The influencing factor mapping table reveals that the effects of ChatGPT on EFL learners are not uniform but contingent on the interaction among learner characteristics, instructional design, and technology use patterns, supporting differentiated rather than uniform approaches to ChatGPT integration in college EFL instruction.

## 1 Introduction

Since its public release in November 2022, ChatGPT has rapidly permeated higher education settings worldwide, with college-level EFL classrooms demonstrating particularly notable rates of adoption. Across courses in comprehensive English, academic writing, translation, and oral communication, ChatGPT has been widely employed as a feedback tool, conversational partner, or content generator (Yang and Li, 2024; Li et al., 2025). Accompanying this trend, a growing body of empirical research has begun to examine changes in learners' affective and cognitive experiences following the integration of ChatGPT into instruction, addressing constructs such as learning motivation, foreign language anxiety, self-efficacy, cognitive load, critical thinking, and learning engagement (Teng, 2024; Klimova et al., 2024). The findings that have emerged present a complex picture. Some studies report gains in motivation, reductions in writing anxiety, and increases in learning engagement (Song and Song, 2023; Du and Alm, 2024), while others document significant rises in cognitive load during the prompt engineering process, learner concerns about over-reliance on AI, and signs of cognitive offloading that may undermine deep learning (Woo et al., 2024; Li et al., 2026). The fact that the same construct yields contradictory results across different studies itself suggests that the factors shaping learner experiences may extend well beyond whether ChatGPT is used.

In recent years, multiple reviews and meta-analyses have systematically synthesized the evidence in this area, reaching a basic consensus on the question of “what effects ChatGPT has on EFL learners.” Systematic reviews have consistently reported positive trends in motivation, anxiety reduction, and wellbeing among EFL learners using generative AI tools (Dong et al., 2026; Jeon, 2025). Meta-analyses pooling experimental comparisons have confirmed positive overall effects on learning outcomes and engagement (Wu et al., 2026; Heung and Chiu, 2025; Saarela et al., 2026). The evidence for higher-order cognitive outcomes is less uniform, with effects on critical and creative thinking varying as a function of instructional design rather than following a single direction (Li et al., 2026). Several of these meta-analyses have further incorporated moderator variable analyses, identifying intervention duration, instructional setting, disciplinary field, and educational level as variables that moderate effect sizes (Saarela et al., 2026; Wu et al., 2026). These contributions provide a solid evidence base for understanding the overall direction of ChatGPT's effects in EFL learning.

However, “what effects have been produced” and “under what conditions these effects arise” represent two distinct levels of inquiry, and the latter has not been adequately addressed in existing reviews. The moderator variables examined in prior meta-analyses are constrained by the pool of quantitative studies available for aggregation, tending toward structural categories such as educational level, intervention duration, and disciplinary field. Although statistically meaningful, these variables offer limited actionable guidance for classroom decision-making: instructors cannot alter the disciplinary field or educational level, but they can modify feedback approaches, scaffolding strategies, or the presence of prompt training. At the same time, a substantial number of qualitative and mixed-methods studies have reported more fine-grained conditional findings. Learners at different levels of metacognitive awareness, for instance, exhibit markedly different behavioral and affective responses when using ChatGPT (Teng, 2025). The depth of cognitive engagement varies across different feedback sources, whether ChatGPT, instructor, or peer (Koltovskaia et al., 2024). Structured instructional integration and unstructured autonomous use lead to measurably different learning experiences (Lai, 2025). These findings remain scattered across individual studies and have not been systematically extracted or categorized in any review. This means that while the field can determine that ChatGPT influences the affective and cognitive experiences of EFL learners, it cannot yet answer for which learners these effects are most pronounced, under what instructional arrangements positive or negative outcomes are most likely to emerge, or through what technology use patterns effects are most likely to be amplified or attenuated.

The Cognitive-Affective Theory of Learning with Media (CATLM) provides a suitable theoretical framework for organizing and analyzing the influencing factors described above, as it integrates cognitive, affective, and individual-difference dimensions within a single media-learning framework that spans learner characteristics, instructional conditions, and technology features. Building on the cognitive theory of multimedia learning, CATLM introduces three extended assumptions directly relevant to the present review (Moreno, 2006; Moreno and Mayer, 2007). The affective mediation assumption holds that motivational and emotional factors influence the learning process by increasing or decreasing the cognitive resources learners invest in a task, providing a theoretical basis for examining how learner anxiety, motivation, and enjoyment moderate the ChatGPT use experience. The metacognitive mediation assumption posits that self-regulatory processes such as planning, monitoring, and evaluation further shape the depth of learner interaction with instructional content, corresponding directly to the examination of how metacognitive awareness and self-regulation function in ChatGPT-assisted learning. The individual differences assumption maintains that learner characteristics including prior knowledge, self-efficacy, and cognitive style moderate the relationship between instructional conditions and learning outcomes, offering a framework for analyzing learner-level variation. CATLM has recently been applied in empirical research on generative AI-assisted EFL writing, confirming its theoretical applicability in this learning context (Zou et al., 2025). By integrating these three assumptions with instructional design and technology use characteristics, the present review constructs a three-tiered analytical framework that organizes potential influencing factors into learner-level, instructional-level, and technology-level categories to guide the extraction and classification of these factors from the literature.

In this review, the term “influencing factors” is adopted as an umbrella concept encompassing variables identified through statistical mediation or moderation, quasi-experimental condition comparisons, and qualitative thematic analysis. Where the evidence permits, specific findings are described using precise terms such as “mediating pathway” or “conditional comparison.” Although ChatGPT is the primary tool examined in the included studies, this review also incorporates studies using other generative AI tools built on the same large language model architecture, given their shared interaction modes and overlapping instructional functions. Accordingly, this scoping review aims to systematically map the influencing factors reported in empirical research as shaping the affective and cognitive experiences of college EFL learners when using ChatGPT. Three research questions guide the review. RQ1 asks what learner-level factors have been reported in empirical studies as influencing the affective and cognitive experiences of college EFL learners using ChatGPT. RQ2 asks what instructional-level factors have been reported as influencing these experiences. RQ3 asks what technology-use-level factors have been reported as influencing these experiences. By shifting the analytical focus from what effects ChatGPT produces to the conditions under which these effects arise, this review seeks to provide an evidence map that informs differentiated integration of ChatGPT in college English instruction.

## 2 Method

### 2.1 Methodological framework

This study adopted a scoping review methodology, following the five-stage framework proposed by Arksey and O'malley (2005) (identifying the research question, identifying relevant studies, selecting studies, charting the data, and collating and reporting the results) and incorporating the methodological refinements recommended by Levac et al. (2010) and Peters et al. (2021). Reporting adhered to the PRISMA-ScR guidelines (Tricco et al., 2018). No review protocol was registered. The goal of this study was to map the breadth and types of influencing factors reported in the existing literature rather than to quantify specific effect sizes. The included studies encompassed quantitative, qualitative, and mixed-methods designs, and considerable heterogeneity existed across studies in terms of intervention approaches, measurement instruments, and outcome variables, rendering statistical pooling inappropriate. The open-ended framework of a scoping review permits systematic extraction and classification of conditional findings across different research designs.

### 2.2 Search strategy

The systematic search covered three academic databases: Web of Science Core Collection, Scopus, and ERIC (accessed via EBSCOhost). PsycINFO was not included as a separate source because Scopus provides substantial coverage of psychology and educational psychology journals, and the combination of Web of Science, Scopus, and ERIC has been widely used in scoping reviews in applied linguistics and language education. The search strategy was built around four groups of core concepts, connected by the Boolean operator AND. The first group comprised tool-related terms, including ChatGPT, Chat GPT, GPT-4, GPT-4o, GPT-3.5, generative AI, GenAI, and large language model along with their variants. The second group consisted of language learning context terms, including EFL, English as a foreign language, English language learning, ESL, English as a second language, second language, and L2. The third group contained educational level terms, including higher education, university, college, tertiary, undergraduate, and postgraduate along with their variants. The fourth group covered affective and cognitive construct terms, including motivation, anxiety, self-efficacy, emotion, affective, enjoyment, wellbeing, wellbeing, cognitive load, critical thinking, engagement, self-regulation, self-regulated learning, metacognitive, and autonomy along with their variants. The search timeframe was restricted to the period from November 2022 (the public release of ChatGPT) through June 2026, and the final search was executed on July 8, 2026 (Supplementary Table 1). The initial search returned 322 records from Web of Science, 450 from Scopus, and 57 from ERIC, totaling 829 records across the three databases. All search results were imported into EndNote for deduplication, and after removing 328 duplicate records, 501 unique records proceeded to the title and abstract screening stage. In addition to the database search, forward and backward citation tracking of the reference lists of all finally included studies was conducted through Google Scholar to capture potentially missed relevant literature.

### 2.3 Inclusion and exclusion criteria

Four inclusion criteria were applied. (1) The study was empirical in design, encompassing quantitative, qualitative, and mixed-methods approaches. (2) Participants were EFL or ESL learners at the university or higher education level. (3) ChatGPT (any version) or a generative AI tool based on large language models was used as a learning tool in the study. Studies using other LLM-based generative AI tools were included when the tools served comparable instructional functions in EFL learning contexts, given the shared underlying architecture of large language models. (4) The study results contained conditional analyses of affective or cognitive experiences, manifested as subgroup comparisons by learner characteristics, task type, or usage mode, mediation or moderation analyses, or qualitative themes describing the influence of individual or contextual differences on affective and cognitive experiences.

Five exclusion criteria were applied. (1) Non-empirical publications, such as theoretical discussions, reviews, editorials, book reviews. Conference abstracts were also excluded due to insufficient methodological detail for full assessment. (2) Studies with K-12 participants. (3) Studies that reported only the main effects of ChatGPT use without any conditional analysis or description of influencing factors. (4) Studies focused exclusively on teacher experiences without addressing learner experiences. (5) Studies involving other AI tools that did not include ChatGPT or generative AI tools based on large language models. The search was limited to publications written in English.

Screening proceeded in two stages. During the title and abstract screening stage, the author screened all deduplicated records against the criteria listed above, excluding studies that clearly did not meet the inclusion criteria. During the full-text screening stage, studies that passed title and abstract screening underwent full-text assessment to confirm whether all four inclusion criteria were satisfied. The screening process was managed using the Rayyan online tool.

### 2.4 Data extraction and analysis

Data were extracted from all included studies using a pre-designed standardized extraction form. Extraction fields included author(s) and year, country or region, research design and methods, sample size and learner characteristics, ChatGPT version or generative AI tool used, course type, dependent variable type (affective, cognitive, or both), influencing factors reported in the study, the analytical level to which each factor was assigned (learner level, instructional level, or technology level), and the reported direction (positive facilitation, negative inhibition, non-significant, or complex interaction). The author completed all data extraction. Each extracted entry was checked against the original study to ensure accuracy.

Data analysis followed a narrative synthesis approach. During open coding, the author read the results and discussion sections of each included study, extracting all factors reported as influencing affective or cognitive experiences on a case-by-case basis and retaining the original terminology from each study as initial codes (e.g., “AI self-efficacy,” “technology self-efficacy,” “writing self-efficacy”). During axial coding, the initial codes were compared and grouped, merging terms that pointed to the same construct into superordinate categories (e.g., the three codes above were merged into “self-efficacy”) and assigning each category to the learner level, instructional level, or technology level in accordance with the three-tiered analytical framework derived from CATLM. The number and labels of the final categories were data-driven and not predetermined. An influencing factor mapping table was constructed to summarize the number of studies addressing each factor, the reported directions, and the degree of consistency across studies. The degree of cross-study consistency was classified into four categories. “Consistent” was assigned when all studies examining a factor reported effects in the same direction. “Mostly consistent” was assigned when the majority of studies converged in direction but one or more studies reported partial exceptions or additional complexities. “Partially consistent” indicated that studies reported mixed directions or that convergence was observed only under specific conditions. “Limited evidence” was assigned when only one or two studies addressed the factor, precluding assessment of cross-study consistency. Where findings from different studies suggested conditional relationships between factors at different analytical levels, cross-level interaction patterns were also noted.

## 3 Results

### 3.1 Overview of included studies

Following the systematic search and screening process (Figure 1), 26 empirical studies were ultimately included. In terms of publication year, 2 studies were published in 2023, 10 in 2024, 7 in 2025, and 7 in 2026. Regarding geographic distribution, mainland China contributed the largest share (11 studies), followed by Macau, China (3 studies, all from the Teng research series), the Middle East (3 studies, distributed across Saudi Arabia, Iran, and Oman), and South and Southeast Asia (3 studies, distributed across Vietnam, Indonesia, and Bangladesh). Hong Kong, China and Taiwan, China each contributed 1 study, as did New Zealand, Slovakia, Morocco, and the United States.

**Figure 1:**
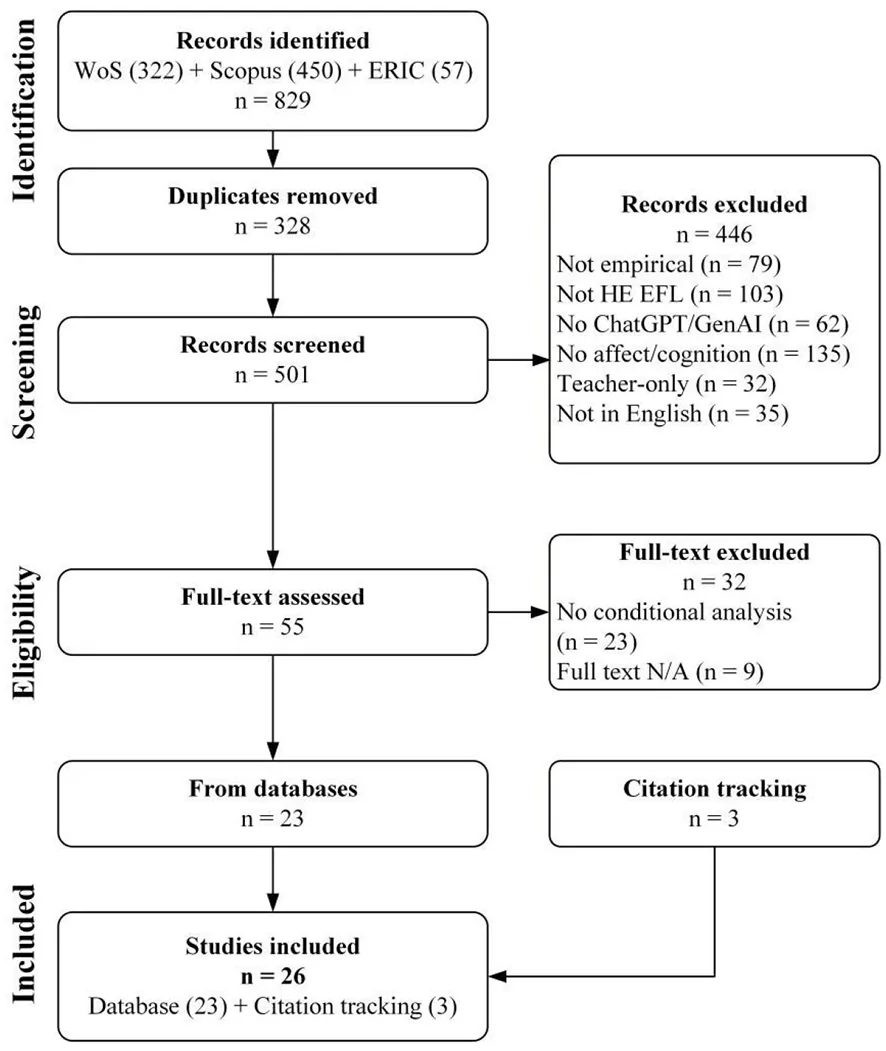
PRISMA flow diagram of the study selection process.

With respect to research design, 11 studies were quantitative (8 using SEM or PLS-SEM and 3 using quasi-experimental or correlational designs), 11 employed mixed methods, and 4 were qualitative (Table 1). Sample sizes ranged from 5 participants (small-scale qualitative studies) to 1,462 (large-scale survey research). Writing courses constituted the predominant course type (16 studies), followed by comprehensive English (3 studies), informal digital learning (2 studies), and mixed course types (2 studies). Speaking, English for academic purposes, and project-based English each accounted for 1 study. ChatGPT was the primary tool used (explicitly identified in 20 studies). Of the remaining 6 studies, 2 examined broader AI tools that explicitly included ChatGPT (No. 4, 22), and 4 employed other LLM-based generative AI tools serving comparable instructional functions without specifying ChatGPT as the tool used (No. 19, 24, 25, 26).

**Table 1:** Characteristics of included studies (*N* = 26).

No.	Author(s) and Year	Country/region	Design	*N*	AI tool	Course type	Affective/cognitive outcome(s)	Influencing factor(s)	Factor level
1	Xiao and Yuying (2023)	China	Qualitative (interview)	5	ChatGPT	General English	Critical judgment, perceived learning value	Individual variation in critical evaluation and prompt modification ability	Learner; Technology
2	Song and Song (2023)	China	Mixed methods (quasi-experimental + interview)	50	ChatGPT	Academic writing	Writing motivation, engagement	Instructional mode (AI-assisted vs. traditional); over-reliance concerns in qualitative data	Instructional; Learner
3	Bouzar et al. (2024)	Morocco	Quantitative (correlational)	148	ChatGPT	Academic writing	Writing self-efficacy, technological dependency	Self-efficacy level predicts usage duration; usage frequency predicts reliance (habituation effect)	Learner
4	Chen et al. (2024)	China	Quantitative (SEM)	429	AI tools incl. ChatGPT	Multiple	AI self-efficacy, AI anxiety, attitude, actual use	Self-efficacy → anxiety (–) → attitude (+) → use; serial mediation paths	Learner
5	Du and Alm (2024)	New Zealand	Qualitative (interview, thematic analysis)	24	ChatGPT	EAP (postgraduate)	Autonomy, competence, relatedness	SDT need satisfaction varies by individual; relatedness unmet for some learners	Learner
6	Ghafouri et al. (2024)	Iran	Mixed methods (quasi-experimental)	12T + 48L	ChatGPT	EFL writing	Teacher self-efficacy, learner writing skill	Structured ChatGPT-based instructional protocol vs. conventional; protocol as enabling condition	Instructional
7	Koltovskaia et al. (2024)	United States	Qualitative (stimulated recall, interview)	6	ChatGPT	Academic writing (graduate)	Behavioral, cognitive, affective engagement	Individual variation in cognitive engagement depth and verification behavior	Learner
8	Liu and Ma 2024)	China	Quantitative (SEM)	405	ChatGPT	Informal IDLE	Perceived usefulness, attitude, intention, actual use	Learning context (informal/out-of-class); PU fully mediates PEU → attitude	Instructional
9	Polakova and Ivenz (2024)	Slovakia	Mixed methods (quasi-experimental + focus group)	110	ChatGPT	EFL writing	Writing proficiency, perception of ChatGPT	Qualitative divergence in student perceptions across focus groups	Learner
10	Teng (2024)	Macau, China	Mixed methods (questionnaire + interview)	45	ChatGPT	EFL writing	Writing motivation, self-efficacy, engagement, collaborative tendency	Perceived benefits vs. perceived risks divergence in qualitative data	Learner
11	Woo et al. (2024)	Hong Kong, China	Mixed methods (questionnaire + think-aloud)	21	ChatGPT	EFL writing	Motivation, cognitive load, satisfaction	Prompt engineering as source of high cognitive load; scaffolding need	Technology
12	Xu and Thien (2024)	China	Quantitative (PLS-SEM)	432	ChatGPT	General English	Perceived enjoyment, intention to use	Perceived enjoyment mediates effort/performance expectancy and social influence → intention	Learner
13	Bristi et al. (2025)	Bangladesh	Quantitative (SEM)	159	ChatGPT	Academic writing	Cognitive attitude, affective attitude, behavioral intention	Relative strength of cognitive vs. affective predictors; affective attitude as stronger predictor and moderate mediator	Learner
14	Lai (2025)	Taiwan, China	Quasi-experimental (mixed methods)	89	ChatGPT	Oral English	Speaking anxiety, confidence, metacognitive dialogue	Structured BOPPPS integration vs. unstructured AI practice; structured → lower anxiety, higher confidence	Instructional
15	Liu et al. (2025)	China	Mixed methods (SEM + interview)	867	ChatGPT, Bing Chat	Informal IDLE	Perceived usefulness, intention, actual use	Usage role (tutor vs. conversation partner) emergent from qualitative data; informal learning context	Technology; Instructional
16	Nguyen and Doan (2025)	Vietnam	Mixed methods (quasi-experimental)	50	ChatGPT	PBL English	Learner autonomy, self-efficacy	Facilitation mode (ChatGPT vs. teacher-led PBL); ChatGPT group higher autonomy gains, equal SE gains	Instructional
17	Teng (2025)	Macau, China	Mixed methods (questionnaire + interview)	40	ChatGPT	EFL writing	Motivation, engagement, self-efficacy, collaborative tendency	Metacognitive awareness level (high vs. low MA); high MA → strategic use, low MA → surface copying	Learner
18	Zare et al. (2025)	Oman/Iran	Mixed methods (quasi-experimental + interview)	69	ChatGPT 4.0	Argumentative writing	Task motivation	Motivation trajectory over time (immediate increase, delayed decline); learner-attributed causes (progress, support, enjoyment, individualized feedback)	Learner
19	Zou et al. (2025)	China	Quasi-experimental	60	Self-developed GenAI chatbot	EFL writing	Cognitive load, negative emotions, writing coherence	Feedback format (visualized vs. non-visualized); visualized → lower cognitive load and less negative emotion	Technology
20	Algobaei and Alzain (2026)	Saudi Arabia	Mixed methods (expert evaluation + student survey)	32	ChatGPT	EFL writing	Clarity, usefulness, motivation	Prompt type (general vs. grammar-focused vs. EFL-tailored); EFL-tailored rated highest	Technology
21	Awalia Putri et al. (2026)	Indonesia	Qualitative (descriptive + thematic analysis)	10	ChatGPT	Academic writing	Functional dependency, cognitive dependency	Dependency type differentiation (functional > cognitive); over-reliance awareness	Learner
22	Shan et al. (2026)	China	Quantitative (SEM)	677	LLMs incl. ChatGPT	General English	Technology self-efficacy, enjoyment, anxiety, intention	Self-efficacy → enjoyment (+)/anxiety (–) → PU/PEU → attitude → intention; serial mediation	Learner
23	Teng and Huang (2026)	Macau, China	Quantitative (quasi-experimental)	174	ChatGPT	EFL writing	Affective engagement, behavioral engagement, writing performance	Feedback source (ChatGPT vs. peer feedback); ChatGPT group higher affective and behavioral engagement	Instructional
24	Xu (2026)	China	Quantitative (SEM)	386	Generative AI	EFL writing	Self-regulated learning, learning engagement	Intention → engagement → SRL (chain mediation); engagement as mediating mechanism	Learner
25	Zhang et al. (2026)	China	Quantitative (PLS-SEM)	1,462	GAI tools	Multiple	Learning anxiety, academic stress	Self-efficacy partially mediates GAI support → anxiety (–) and stress (–)	Learner
26	Zhang Y (2026)	China	Quantitative (SEM)	572	AI writing tools	Academic writing	Writing self-efficacy, writing anxiety, writing performance	Technology perception (PU, PEOU, trust) → self-efficacy → anxiety; integrity threat → anxiety (+); trust → anxiety (–) direct and indirect	Learner

### 3.2 Learner-level influencing factors

Of the 26 included studies, 16 reported at least one learner-level factor that served an influencing role in affective or cognitive experiences. The evidence at this level drew on multiple analytical approaches, including statistical mediation in SEM studies, subgroup comparisons in mixed-methods designs, and thematic differentiation in qualitative research. Four categories of influencing factors were identified through inductive coding.

#### 3.2.1 Self-efficacy

Self-efficacy was incorporated into the analysis in 8 studies (No. 3, 4, 10, 12, 17, 22, 25, 26), making it the most frequently reported influencing factor, with a high degree of directional consistency across studies. Multiple large-sample SEM studies collectively revealed mediating pathways through which self-efficacy operated between technology perceptions and affective outcomes. AI self-efficacy negatively predicted anxiety and positively predicted attitude, indirectly facilitating tool use through the dual pathways of reduced anxiety and enhanced attitude (No. 4, *N* = 429). In a larger sample, self-efficacy partially mediated the negative relationships between generative AI support and both learning anxiety and academic stress (No. 25, *N* = 1,462). Technology self-efficacy also transmitted its effects to usage intention through two affective pathways, perceived enjoyment and technology anxiety, which in turn operated through perceived usefulness and perceived ease of use, forming a serial mediation chain (No. 22, *N* = 677). In the context of AI-assisted academic writing, positive perceptions of tool credibility indirectly reduced writing anxiety through writing self-efficacy, while perceived academic integrity threat directly and positively predicted anxiety, with the two pathways jointly acting on the same affective outcome (No. 26, *N* = 572). Perceived enjoyment, as an intrinsic motivational variable, mediated the effects of effort expectancy and social influence on usage intention (No. 12, *N* = 432).

The consistency of these quantitative pathways was echoed but also complicated by qualitative and mixed-methods findings. Learners who reported gains in self-efficacy simultaneously expressed concerns that technology dependence might erode their autonomous writing ability (No. 10, *N* = 45). In an academic writing context, growth in writing confidence was observed alongside a concurrent increase in ChatGPT reliance (No. 3, *N* = 148). This coexistence of efficacy and dependency suggests that the protective role of self-efficacy in ChatGPT-assisted EFL learning is not unidirectional. When the source of efficacy beliefs is partly built on tool-assisted performance, the sustainability of those beliefs may be undermined by developing dependency tendencies.

#### 3.2.2 Metacognitive awareness and self-regulation

Three studies incorporated metacognition-related constructs into their analyses (No. 17, 24, 18). The study that directly grouped participants by metacognitive awareness level provided the clearest conditional comparison (No. 17, *N* = 40): the high metacognitive awareness group scored significantly higher on motivation, engagement, self-efficacy, and collaborative writing tendency, and the two groups diverged qualitatively in their ChatGPT feedback use behavior, with the high group engaging in strategic utilization and critical evaluation while the low group tended toward direct copying of outputs. In a mediation model of self-regulated learning, learning engagement mediated the relationship between usage intention and self-regulated learning (No. 24, *N* = 386), suggesting that the development of self-regulation capacity is not a direct consequence of technology use but is instead realized indirectly through the depth of engagement. The temporal trajectory of motivation was also linked to metacognitive processes: learners attributed part of their post-intervention motivation gains to the development of metacognitive awareness, specifically to recognizing, through ChatGPT feedback, specific problems in their writing that they had not previously noticed (No. 18, *N* = 69). Taken together, the three studies point to a common pattern in which metacognitive level determines whether learners can transform ChatGPT from a passive answer source into an active learning scaffold.

#### 3.2.3 Motivation orientation and affective experience

Five studies reported that motivation and affective states played influencing roles in the ChatGPT use experience (No. 2, 5, 12, 13, 18). Within the SDT framework, ChatGPT's satisfaction of autonomy and competence needs was relatively consistent across learners, but the satisfaction of relatedness needs showed a split: some learners perceived a sense of companionship, while others felt a sense of detachment due to reduced interpersonal interaction (No. 5, *N* = 24). Both cognitive attitude and affective attitude significantly predicted usage intention, yet affective attitude emerged as the stronger predictor, and the two attitudes mutually mediated each other (No. 13, *N* = 159), indicating that the formation of ChatGPT usage intention is simultaneously driven by rational evaluation and emotional experience. Motivational change exhibited temporal conditionality, rising significantly after the intervention but declining at the delayed posttest, with learners attributing the initial motivational gains to perceived skill improvement, the supportive environment provided by ChatGPT, enjoyment during interaction, and metacognitive awareness prompted by individualized feedback (No. 18, *N* = 69). ChatGPT-assisted instruction produced significantly greater improvements in writing motivation than traditional instruction (No. 2, *N* = 50), though qualitative data simultaneously revealed concerns among some learners about over-reliance.

#### 3.2.4 Dependency tendency and critical judgment

Four studies reported that the degree of learner dependency and the capacity for critical evaluation functioned as factors influencing cognitive experience (No. 1, 3, 7, 21). Functional dependency (surface-level processing such as grammar correction, vocabulary improvement, and sentence construction) was more prevalent than cognitive dependency (deeper processing such as idea generation and argument development), and learners themselves demonstrated awareness of the risk of over-reliance (No. 21, *N* = 10). The coexistence of self-efficacy and dependency was corroborated in another study, where gains in writing confidence were accompanied by a habituation effect in which usage frequency predicted the degree of reliance (No. 3, *N* = 148). Regarding critical judgment, individual differences existed in learners' capacity to evaluate ChatGPT output. Some learners proactively modified their prompts to optimize output quality, while others tended to accept suggestions without scrutiny (No. 1, *N* = 5). Graduate students using ChatGPT to revise academic texts generally displayed high levels of cognitive engagement, but their responses when questioning feedback accuracy diverged into two modes: active verification and passive acceptance (No. 7, *N* = 6).

### 3.3 Instructional-level influencing factors

Nine studies reported instructional-level influencing factors (No. 2, 6, 8, 9, 14, 15, 16, 18, 23), from which three categories were identified through inductive coding.

#### 3.3.1 Feedback source and facilitation mode

Differences in feedback source and instructional facilitation mode were examined in 5 studies (No. 6, 9, 16, 18, 23). In a quasi-experimental comparison between a ChatGPT feedback group and a peer feedback group, the ChatGPT group exhibited greater gains in both affective and behavioral engagement, and a significant between-group difference in writing performance also emerged (No. 23, *N* = 174). ChatGPT-facilitated project-based learning and teacher-facilitated project-based learning produced divergent outcomes: the former yielded greater gains in learner autonomy, while the two groups achieved comparable improvements in self-efficacy. Qualitative analysis further revealed that ChatGPT facilitation promoted group initiative and experimentation, whereas teacher facilitation reinforced established routines and deference to authority (No. 16, *N* = 50). The integration of ChatGPT into a structured writing instruction protocol positively affected both learner writing skills and teacher self-efficacy, though this outcome was contingent on the presence of a structured protocol as a precondition (No. 6, 12T + 48L). Learners who completed writing tasks through interaction with ChatGPT attributed their motivation gains to individualized feedback and a supportive interactional environment (No. 18, *N* = 69). Affective receptivity toward ChatGPT feedback was not uniformly positive; focus group discussions surfaced reservations concerning feedback accuracy and a perceived lack of personal warmth (No. 9, *N* = 110). Collectively, these findings indicate that the impact of feedback source on the learning experience depends on how feedback is integrated into the instructional design and on learners' affective receptivity toward the feedback source, rather than on the technical attributes of the source *perse*.

#### 3.3.2 Degree of instructional structuring

The contrast between structured integration and unstructured use was examined in 3 studies (No. 2, 6, 14), all of which pointed in the same direction. Learners who received structured instruction within the BOPPPS framework demonstrated significantly greater improvement across multiple speaking skills compared to those in the unstructured AI practice group. Qualitative analysis revealed that structured integration repositioned ChatGPT from a static tool to a metacognitive dialogue partner, reducing speaking anxiety and fostering more autonomous and confident language use (No. 14, *N* = 89). The experimental group receiving ChatGPT-assisted writing instruction significantly outperformed the traditional instruction control group in both writing skills and motivation (No. 2, *N* = 50). The conditional role of a structured instructional protocol in shaping outcomes was likewise supported (No. 6). The consistent direction across all three studies suggests that the instructional effectiveness of ChatGPT depends to a considerable extent on how it is embedded within the instructional design, and that unrestricted use without structural support fails to yield equivalent affective and cognitive benefits.

#### 3.3.3 Learning context

Contextual differences between formal in-class learning and informal out-of-class learning were reported in 2 studies (No. 8, 15). In an informal digital English learning context outside the classroom, learner adoption of ChatGPT followed a trajectory from trial use to routine use, and two distinct roles, “tutor” and “conversation partner,” emerged naturally during the process (No. 15, *N* = 867). In the out-of-class context, perceived usefulness fully mediated the effect of perceived ease of use on attitude, and behavioral intention demonstrated strong predictive power for actual use behavior (No. 8, *N* = 405). In contrast to in-class studies where learner experiences were primarily shaped by instructor design and task requirements, experiences in informal contexts were molded more by learners' own usage motivation and exploratory behavior.

### 3.4 Technology-use-level influencing factors

Six studies reported technology-use-level influencing factors (No. 1, 11, 15, 19, 20, 21), from which three categories were identified through inductive coding.

Prompt strategy and design. Three studies reported on the impact of prompt design and usage patterns on the learning experience (No. 1, 11, 20). A direct comparison of three prompt types showed that EFL-tailored prompts received the highest ratings in both expert evaluation and student perception, with learners finding this type of feedback easier to comprehend and more conducive to motivation (No. 20, *N* = 32). The influence of prompts extended beyond output quality: the prompt engineering process itself constituted a source of cognitive load, as learners reported substantial cognitive demands when designing and revising prompts, particularly in the absence of scaffolding (No. 11, *N* = 21). Individual differences in learners' ability to modify prompts to optimize output directly affected the extent to which they obtained effective feedback (No. 1, *N* = 5). This ability difference simultaneously involved learner-level critical judgment and technology-level prompt quality, exhibiting cross-level interactive characteristics.

Feedback presentation format. One study directly manipulated feedback presentation format as an experimental variable (No. 19). A quasi-experimental design grounded in CATLM found that learners who received visualized feedback (color changes, tabular formatting, bolded text) achieved significantly greater improvement in writing coherence than the text-only feedback group, while also reporting lower levels of negative emotion and reduced cognitive load (No. 19, *N* = 60). The presentation format of AI-generated content can influence learners' affective and cognitive experiences independently of the feedback content itself, though this conclusion currently rests on a single study and requires further replication.

Usage role and mode. Two studies reported on the effects of the roles learners assigned to ChatGPT on their experiences (No. 15, 21). In an informal learning context, learner use of ChatGPT naturally differentiated into two roles, “tutor” and “conversation partner,” with differences in learning experience and cognitive engagement depth observed between the two (No. 15, *N* = 867). The differentiation between functional dependency and cognitive dependency reflected the impact of usage mode on the level of cognitive engagement: learners who used ChatGPT for surface-level linguistic processing and those who used it for deeper content generation displayed distinct profiles of dependency and cognitive risk (No. 21, *N* = 10).

### 3.5 Influencing factor mapping

Evidence density across the three levels exhibited a clear gradient (Table 2). Learner-level evidence was supported by the largest body of research (16 studies, 4 factor categories), with self-efficacy demonstrating consistent mediating pathways across 8 SEM and mixed-methods studies. Instructional-level evidence was concentrated in feedback source and facilitation mode (5 studies) and degree of structuring (3 studies), while learning context was addressed in only 2 studies. Technology-level evidence was the most limited, with feedback presentation format supported by only 1 direct experimental study and the analysis of usage roles relying primarily on qualitative findings.

**Table 2:** Influencing factor mapping table.

Factor level	Influencing factor	Studies (No.)	*N* of Studies	Direction	Consistency
Learner	Self-efficacy	3, 4, 10, 12, 17, 22, 25, 26	8	Higher SE → lower anxiety, more positive affect, stronger intention	Consistent
	Metacognitive awareness/self-regulation	17, 18, 24	3	Higher MA → deeper engagement, strategic use	Consistent
	Motivation orientation/affective attitude	2, 5, 12, 13, 18	5	Intrinsic motivation/enjoyment → stronger engagement; temporal decay observed	Mostly consistent
	Dependency tendency/critical judgment	1, 3, 7, 21	4	Higher dependency → surface processing; critical judgment varies individually	Consistent
Instructional	Feedback source/facilitation mode	6, 9, 16, 18, 23	5	ChatGPT → higher engagement/autonomy; affective acceptance mixed	Partially consistent
	Structured vs. unstructured integration	2, 6, 14	3	Structured → better outcomes, lower anxiety	Consistent
	Learning context (formal vs. informal)	8, 15	2	Informal → more autonomy-driven, exploratory use	Limited evidence
Technology	Prompt strategy/type	1, 11, 20	3	Tailored prompts → better perception; prompt engineering → higher cognitive load	Partially consistent
	Feedback presentation format	19	1	Visualized → lower cognitive load, less negative emotion	Limited evidence
	Usage role/mode	15, 21	2	Tutor vs. partner → different engagement; functional vs. cognitive dependency	Limited evidence

Cross-level interactions were suggested by patterns observed across the data. No single study simultaneously measured variables across these levels, and these interaction patterns are therefore inferred from converging findings across separate studies and remain to be directly tested. Learners with high self-efficacy appeared able to maintain positive experiences even under conditions of limited structural support, whereas those with low self-efficacy relied more heavily on structured instructional design to reduce anxiety (No. 4, 14, 22). Learners with high metacognitive awareness were more inclined to proactively modify prompts for better output, achieving deeper cognitive engagement, while those with low metacognitive awareness were more likely to remain at the surface level of use when prompt training was absent (No. 1, 11, 17).

## 4 Discussion

### 4.1 Theoretical interpretation of key findings

The gradient in evidence density across the three levels reflects the current research emphasis in this field. Learner-level influencing factors far exceed instructional-level and technology-level factors in both quantity and consistency, and CATLM is precisely the theory that places individual differences at the center of its framework (Moreno, 2006; Park et al., 2014). Conditional analysis of instructional conditions and technology characteristics remains notably underrepresented in the current literature.

The mediating pathways of self-efficacy display a multi-channel structure. Across different studies, the same construct exerts its mediating role through distinct affective channels, including anxiety, attitude, enjoyment, and academic stress. Technology trust and perceived academic integrity threat simultaneously act on writing anxiety through pathways that run in opposite directions. In traditional multimedia learning environments, affective activation is relatively passive, with learners generating emotional responses after receiving information. The dialogic interaction afforded by ChatGPT continuously triggers evaluative cognitive processes: each round of interaction involves judgments about tool credibility, output accuracy, and compliance with academic norms, and different judgments activate different affective channels. The affective mediation assumption of CATLM, when applied to this environment, needs to be understood as a multi-pathway parallel process rather than a linear moderation of cognitive processing by a single affective variable.

The coexistence of efficacy beliefs and dependency tendencies poses a shared theoretical challenge for both CATLM and SDT. Both theories assume that a sense of competence originates from learners' own skill development. When the source of competence beliefs is partly grounded in external tool assistance, two cognitive appraisals may coexist: “my writing has improved” and “could I still write well without ChatGPT.” University students' dependency on ChatGPT has been found to differentiate into multiple profiles (Stojanov et al., 2024), and high-frequency use of AI tools may negatively affect critical thinking through cognitive laziness and metacognitive weaknesses (Yurt and Kuşci, 2025), while compulsive use may trigger psychological distress through loneliness and social avoidance (Duong et al., 2025). Positive affective experiences and potential cognitive risks develop in parallel, both influenced by self-efficacy level, though through different directions and mechanisms. The differentiation between functional dependency and cognitive dependency further indicates that different levels of use correspond to different cognitive risks.

Metacognitive awareness and self-efficacy serve complementary functions. Self-efficacy shapes learners' confidence and affective responses when using ChatGPT, whereas metacognitive awareness shapes usage patterns and the depth of cognitive engagement. The interaction between these two constructs may constitute a dual threshold for effective ChatGPT-assisted learning, though this possibility is based on converging patterns across separate studies and has not been directly tested within a single study.

Among the instructional-level findings, the feedback source effect reveals a dissociation between cognitive efficiency and affective receptivity. The informational advantages of ChatGPT feedback do not automatically translate into affective acceptance. Teachers also hold dual perceptions of ChatGPT feedback (Guo and Wang, 2024), and their own affective and cognitive responses may indirectly shape learner experiences by influencing how ChatGPT is integrated into classroom practice (Wang et al., 2026). Cognitive efficiency and affective safety may need to be safeguarded through distinct instructional design strategies.

Technology-level evidence, though the smallest in volume, points to a direction for theoretical extension of CATLM. The original CATLM definition of “media characteristics” centers on the modality and presentation of information, designed for static multimedia materials. The “media characteristics” of ChatGPT expand to encompass new parameters such as prompt design, output format, and interaction role. The CASTLE theory has already extended CATLM by adding a social processing dimension (Schneider et al., 2022), and the findings of this review suggest that further extension toward interactive AI learning environments should also incorporate human-computer interaction design parameters into the framework.

### 4.2 Practical implications for college english instruction

The conditional effects of learner-level influencing factors imply that the integration of ChatGPT into college English courses requires a tiered design rather than uniform implementation. For learners with lower self-efficacy or weaker metacognitive awareness, providing usage guidance, task decomposition, and feedback interpretation scaffolding alongside the introduction of ChatGPT represents a necessary condition for reducing affective risk and promoting deeper engagement. Embedding metacognitive training within ChatGPT-assisted instructional workflows, such as reflective writing journals, explicit instruction in revision strategies, and exercises in evaluating ChatGPT output, may help narrow the experience gap across learners with different profiles.

The finding that ChatGPT yields markedly better outcomes when embedded within a designed instructional framework than when used through unstructured autonomous exploration provides preliminary guidance for instructional practice, though this pattern is based on three studies and requires further replication across a wider range of instructional contexts. Within the context of college English curriculum reform, transforming ChatGPT from an extracurricular auxiliary tool into an organic component of course design requires instructors to engage in systematic planning of task design, timing of use, and feedback integration methods. A blended feedback model, in which ChatGPT provides initial feedback while the instructor delivers higher-order commentary and affective support, may be better positioned than single-source feedback to address both cognitive and affective dimensions simultaneously. The instructor's role in this process shifts toward that of a guide for AI use and an integrator of feedback (Kasneci et al., 2023; Al-Zahrani, 2025). The low-anxiety practice environment afforded by AI-assisted interaction in speaking courses has received preliminary empirical support (Fathi et al., 2024), and the realization of its benefits likewise depends on structured instructional design.

Prompt design competence needs to be incorporated into courses as explicit instructional content rather than treated as a skill learners can develop on their own. Across different course types, including comprehensive English, academic writing, and translation, the specific forms of prompt strategies should be tailored to match the linguistic and cognitive demands of different tasks.

### 4.3 Limitations and future research directions

This review did not conduct methodological quality assessment of included studies. The 26 included studies varied substantially in sample size (from 5 to 1,462 participants) and research design (from qualitative interviews to large-scale SEM surveys), and the narrative synthesis did not differentiate among these variations. The self-efficacy mediating pathways supported by 8 SEM studies and the feedback presentation effect supported by a single quasi-experiment differ fundamentally in inferential strength, yet both are presented in a comparable manner in the mapping table. Conducting systematic reviews and meta-analyses of influencing factors for which the evidence base is relatively well documented would enable graded evaluation of the evidence beyond what the present mapping provides.

All screening, data extraction, and coding were conducted by the sole author. A standardized extraction form and systematic cross-checking against the original studies were employed to maintain consistency, but the review would have benefited from independent dual verification to further reduce the possibility of selection and coding bias.

The tool scope was restricted to ChatGPT and generative AI tools based on large language models; studies using other types of AI tools were excluded (Xiao et al., 2026). The inclusion of studies using broader LLM-based generative AI tools alongside ChatGPT-specific studies may introduce a degree of heterogeneity, as differences in interface design, model capability, and user experience across tools could affect the generalizability of the findings. The search language was limited to English, leaving out a substantial volume of Chinese-language empirical research from mainland China. The use of predefined affective and cognitive search terms may also have excluded relevant studies that examined related psychological constructs under different terminology. The geographic concentration of the included studies, with 22 of 26 originating from Asia, limits the generalizability of findings to other cultural contexts.

The extraction of influencing factors depended on the reporting depth of the original studies. The inclusion criteria required that studies contain conditional analyses, and those reporting only main effects were excluded. The mapping table therefore represents the influencing factors that researchers chose to examine and report, constrained by the design choices and analytical strategies of the original investigators. The predominance of writing courses among included studies (16 of 26) reflects the current research preference in this field rather than a search restriction imposed by this review. Both the coverage of the influencing factor mapping table and the evidence density gap across the three levels are bounded by these upstream conditions.

Several patterns identified in this review are constrained by the design characteristics of the existing evidence. The coexistence of efficacy beliefs and dependency tendencies has been observed in multiple cross-sectional studies, but whether the two develop in parallel or follow a temporal sequence cannot be determined from cross-sectional data. The decline in motivational gains at delayed posttesting has been addressed in only a single study, and existing meta-analyses have similarly noted that current evidence provides insufficient tracking of long-term effects (Deng et al., 2025). The evidence density gap across the three levels means that the mapping table offers narrower coverage at the instructional and technology levels, and conclusions drawn from these two levels will need to be revisited as more empirical evidence accumulates. Cross-level interaction patterns, such as the interaction between self-efficacy and degree of structuring or between metacognitive awareness and prompt strategy, are currently based solely on indirect inference across studies; evidence from single studies that simultaneously manipulate or measure these interactions does not yet exist.

## 5 Conclusion

Existing reviews have established the direction of ChatGPT's effects on the affective and cognitive experiences of college EFL learners. Building on that foundation, this review turns to the conditional question of under what circumstances these effects arise. Through systematic mapping of 26 empirical studies examining ChatGPT and closely related LLM-based generative AI tools, 10 types of influencing factors distributed across the learner, instructional, and technology levels were identified and presented in an influencing factor mapping table that documents the evidence density, effect direction, and cross-study consistency for each factor. The evidence gradient across the three levels indicates that the conditional role of learner-level characteristics, particularly self-efficacy and metacognitive awareness, has been supported by a relatively substantial body of empirical work, while investigation into how instructional design and technology use patterns shape learning experiences remains at an early stage.

The multi-channel mediating structure of self-efficacy and the coexistence of efficacy beliefs with dependency tendencies together suggest that positive affective experiences and potential cognitive risks in ChatGPT-assisted EFL learning may develop along parallel tracks. Structured instructional integration exhibited the most consistently positive effects among all influencing factors, offering the most well-supported guidance for instructional practice among the factors examined. Preliminary findings on prompt strategy and feedback presentation format open a technology design space that is still in its infancy. The practical implications of these findings converge on three directions for action: tiered instructional design, structured course-level integration, and the inclusion of prompt training as curricular content.

The research agenda in this field is shifting from “whether ChatGPT affects EFL learners' affective and cognitive experiences” toward “for whom, under what instructional arrangements, and through what usage patterns does it produce which effects.” The evidence gaps in the mapping table and the density imbalance across the three levels delineate the directions that warrant priority investment in future research.
