# Supplementary material for: When does ChatGPT help or hinder? A scoping review of influencing factors in college EFL learners' affective and cognitive experiences
Source: Front Psychol. 2026 Sep 29;17:1949431. doi: 10.3389/fpsyg.2026.1949431 (PMC13658153; doi:10.3389/fpsyg.2026.1949431)
Supplement: Supplementary file 1 [file Table_1.docx]

Supplementary Material

**Supplementary Table 1.** Complete search strategies for Web of Science, Scopus, and ERIC (search date: July 8, 2026; time span: November 2022 - June 2026).

| Database | Platform | Search query | Date filter | Records |
| --- | --- | --- | --- | --- |
| Web of Science Core Collection | webofscience.com | TS=(“ChatGPT” OR “Chat GPT” OR “GPT-4” OR “GPT-4o” OR “GPT-3.5” OR “generative AI” OR “GenAI” OR “large language model*”) AND TS=(“EFL” OR “English as a foreign language” OR “English language learning” OR “ESL” OR “English as a second language” OR “second language” OR “L2”) AND TS=(“higher education” OR universit* OR college OR tertiary OR undergraduate* OR postgraduate*) AND TS=(motivation OR anxiety OR self-efficacy OR emotion* OR affective OR enjoyment OR “well-being” OR wellbeing OR “cognitive load” OR “critical thinking” OR engagement OR “self-regulat*” OR metacognit* OR autonomy) | 2022-2026 | 322 |
| Scopus | scopus.com | TITLE-ABS-KEY(“ChatGPT” OR “Chat GPT” OR “GPT-4” OR “GPT-4o” OR “GPT-3.5” OR “generative AI” OR “GenAI” OR “large language model*”) AND TITLE-ABS-KEY(“EFL” OR “English as a foreign language” OR “English language learning” OR “ESL” OR “English as a second language” OR “second language” OR “L2”) AND TITLE-ABS-KEY(“higher education” OR universit* OR college OR tertiary OR undergraduate* OR postgraduate*) AND TITLE-ABS-KEY(motivation OR anxiety OR self-efficacy OR emotion* OR affective OR enjoyment OR “well-being” OR wellbeing OR “cognitive load” OR “critical thinking” OR engagement OR “self-regulat*” OR metacognit* OR autonomy) AND PUBYEAR > 2021 AND PUBYEAR < 2027 | PUBYEAR > 2021 AND PUBYEAR < 2027 | 450 |
| ERIC | EBSCOhost (Fudan University Library) | Row 1 (AB): ChatGPT OR “Chat GPT” OR “GPT-4” OR “GPT-4o” OR “GPT-3.5” OR “generative AI” OR “GenAI” OR “large language model” AND Row 2 (AB): EFL OR “English as a foreign language” OR “English language learning” OR ESL OR “English as a second language” OR “second language” OR “L2” AND Row 3 (AB): “higher education” OR university OR college OR tertiary OR undergraduate OR postgraduate AND Row 4 (AB): motivation OR anxiety OR self-efficacy OR emotion OR affective OR enjoyment OR “well-being” OR wellbeing OR “cognitive load” OR “critical thinking” OR engagement OR “self-regulated learning” OR “self-regulation” OR metacognitive OR autonomy | 11/2022-06/2026 | 57 |
| Total |  |  |  | 829 |

*Note. Forward and backward citation tracking was conducted via Google Scholar on the reference lists of all finally included studies. Deduplication and screening were managed using EndNote and Rayyan.*
